# CURB-65 and Long-Term Mortality of Community-Acquired Pneumonia: A Retrospective Study on Hospitalized Patients

**DOI:** 10.7759/cureus.36052

**Published:** 2023-03-12

**Authors:** Pedro Carlos, Ricardo Gomes, Joana Coelho, Catarina Chaves, Célia Tuna, Marlene Louro

**Affiliations:** 1 Internal Medicine Department, Centro Hospitalar Universitário Cova da Beira, Covilhã, PRT; 2 Endocrinology Department, Centro Hospitalar Tâmega e Sousa, Penafiel, PRT

**Keywords:** community acquired pneumonia, retrospective observational study, classification, prognosis, mortality

## Abstract

Background

Community-acquired pneumonia remains a significant factor in global mortality. Several clinical scoring models are used for predicting pneumonia severity and mortality, aiding in the clinical decision relative to the therapeutic approach, including the CURB-65 score. However, currently, no models exist to identify high-risk patients relative to long-term prognosis when recent evidence reveals a significantly higher mortality rate in the first year after community-acquired pneumonia (CAP) hospitalization.

Purpose of the study

The purpose of this study is to evaluate the application of the CURB-65 scoring model in our population and examine its potential to predict prognosis and subsequent mortality 6 months after hospitalization. Other potential factors influencing mortality during and after hospitalization were characterized: patient demographics, nosocomial infections, readmissions, and identified pathogens.

Study design

We conducted a retrospective observational study, enrolling 130 patients admitted with a diagnosis of CAP in the department of internal medicine of Centro Hospitalar Universitário Cova da Beira between January and December of 2018. Consultation of electronic medical records was used to calculate the CURB-65 score on admission at the first hospitalization by CAP, categorizing patients into five risk groups. Mortality and readmission were evaluated after 30, 90, and 180 days.

Key results

High-risk patients (CURB>2) accounted for 96.9% of our study population. Inpatient mortality of 13%, increasing to 21.5% after six months, was similar to previous studies and was correlated to the CURB-65 score on admission. A microbiologic agent was identified in 37% of cases, with 53% isolates of Streptococcus (S.) pneumoniae.

Conclusions

Identifying high-risk patients is important for more individualized healthcare and management. The CURB-65 score, only validated for a short-term (30 days) prediction, demonstrates a potential to also predict mortality and rehospitalization in the six-month period after hospitalization, as supported by our findings and previous studies.

## Introduction

Community-acquired pneumonia (CAP) persists as a ubiquitous clinical entity in medical practice despite continuing advances in its therapeutic and diagnostic approach [1]. It is a major cause of global mortality and morbidity [2-3] and one of the leading causes of death among all infectious diseases [4-5]. It is responsible for an estimated 3,500,000 deaths annually [6], despite accounting for only 5-12% of all cases of lower respiratory tract infections [7].

In Europe, although the overall impact is unclear due to heterogeneity in therapeutic strategies and different admission criteria [2,8], projections refer to 3,370,000 cases every year, with hospitalization rates between 20% and 50% [9]. Inpatient mortality is reported to be around 8% [4], increasing to 14% [9] 90 days after discharge or even as high as 20-28% [4] if admission to an intensive care unit (ICU) is required, which occurs in 10% [6-8] of all patients.

Portuguese data from 2014 reveal a total of 53,230 patients hospitalized by CAP, associated with inpatient mortality and readmission rates of 26% and 12%, respectively [10]. It is estimated that this value represents only 25-50% of total cases per year [11], with several studies showing a recent increasing trend of total hospitalizations and in-hospital mortality, the latter from 17.3% between 1998 and 2000 to 20.4% between 2000 and 2009 [8,12].

Pneumonia, by definition, is characterized by an acute lung infection that occurs when the immune system is unable to neutralize and eliminate a pathogen from the lower airways and alveoli, which often results from microaspiration of oropharyngeal secretions [6]. The more clinically evident signs and symptoms of this pathology, both systemic and respiratory, are mainly due to the organism's inflammatory and immunological response [13]. The maintenance of this pro-inflammatory systemic state, even after clinical resolution, is the main potential factor associated with a higher risk of re-hospitalization and mortality observed in the patients recently hospitalized for CAP compared to the general population [6]. These findings are observed up to three to five years after an episode of CAP [5,14], mainly related to an increase in cardiovascular events [3,14].

Among described pathological agents, Streptococcus pneumoniae assumes a prominent role as the most frequent agent of CAP globally, isolated in 25% of cases [1]. However, current diagnostic methods still result in an unsatisfactory percentage of CAP cases without an identified agent, varying between 46% and 62% in the US [3,15-16] and 35.3% to 67% in Europe [2,17]. Viral co-infection is reported to occur in 20% of cases [3,16].

Stratification by disease severity and prognosis can facilitate the identification of low-risk patients that could receive outpatient treatment, reducing health-associated costs and potential inpatient complications while enabling early recognition of cases requiring admission to the intensive care unit (ICU) [11,18]. With this objective, several severity scales were designed, with CURB-65 and pneumonia severity index (PSI) among the most studied and validated in this context [18-19]. The CURB-65 scale applies five variables that allow stratification into various risk groups but does not value other relevant prognostic factors such as hypoxia or the presence of bilateral pneumonia [18-19].

Studies comparing these two scales in equal populations are inconclusive, hindering consensual conclusions on the superiority, if any, of these two scales [7,18-20]. Both present good discriminatory results for mortality and the capacity to identify patients that require admission to intensive care units [19-20]. Nevertheless, their potential for overestimating mortality serves as an important limitation and are thus only indicated as auxiliary tools to clinical decision.

Contrary to previous national studies [21-22] with the predominant use of PSI, we opted for the application of CURB-65 based on the absence of superiority of any of the scales, its greater easiness of application, and the argument that all the necessary information for its utilization is far more likely to be present in the medical record on admission to the emergency department. We did not find any recently published national study using the CURB-65 scale to compare obtained data and conclusions, nor any previous national study with a similar objective of assessing the potential association between admission score on the CURB-65 scale and long-term mortality beyond the recommended 30 days.

## Materials and methods

The purpose of this study is to evaluate and validate the application of one of the most widely used and studied short-term mortality prediction clinical scores for this pathology (CURB-65), comparing our data with mortality reference values for the different risk subgroups. In addition, we intended to study the correlation between multiple variables: age, gender, CURB-65 classification at admission, frequency of nosocomial infections, and length of hospital stay with readmission and mortality rates during hospitalization and after 30, 90, and 180 days.

With this data, we expect to conclude how the application of the CURB-65 scale in a CAP hospital population allows for a prediction of mortality not only in the first 30 days as originally proposed [18] but also in the long term, as described in subsequent studies [5].

Secondary objectives are to characterize other potential factors associated with in-patients and six-month mortality, such as patient demographics, nosocomial infections, readmissions, and microbiologic agent identification, comparing the distribution of typical and atypical agents to that reported in similar studies [21-22].

We conducted a retrospective observational study with consultation and statistical analysis of the electronic medical records referring to patients with a diagnosis of community-acquired pneumonia that were admitted to the wards of the internal medicine department of the Academic Hospital Center of Cova da Beira between January and December 2018. The following were used as criteria for inclusion in the study: age over 18 years and diagnosis of community-acquired pneumonia supported by clinical, analytical, and imaging evidence. All patients with other diagnoses of other lower respiratory tract infections were excluded. Ultimately, a total of 130 cases were identified and included.

From this sample, demographic information (age, gender) and data relative to the hospitalization episode were collected: microbiological culture identifications, antibiotics administered, total duration of hospitalization, the occurrence of nosocomial infections, and mortality. Methods of microbiological identification were based on blood and sputum cultures, urinary antigen screening, and influenza virus PCR-based methods.

We processed the necessary data present on the medical records to calculate the CURB-65 score (Table 1) at the time of hospitalization, using these results to categorize these patients, according to their predicted mortality risk, in five different risk groups (0-4 points). Mortality and readmission rates were assessed in four selected timeframes on follow-up: during hospitalization and 30, 90, and 180 days after hospitalization on a global sample and between each of the different five risk groups obtained.

**Table 1:** CURB-65 score for pneumonia severity Variables used on CURB-65 calculation and subsequent risk stratification by score on admittance, with a representation of expected mortality and the recommended management of these patients [18]: 0 or 1 points - low mortality risk (1.5%) and outpatient (home) treatment; 2 points - moderate risk (9.2%) and consider hospitalization; 3 points - high risk (22%) and recommended hospitalization; 4 or 5 points - very high risk, severe pneumonia and consider admittance to ICU.

	Description
Confusion	Yes = 1 point or No = 0 points
Urea (Blood urea nitrogen)	> 19 mg/dL = 1 point
Respiratory Rate	> 30 /minute = 1 point
Blood Pressure	Systolic <90 mmHg or Diastolic <60 mmHg = 1 point
-65 age	> 65 years = 1 point

All data were randomized, with a respective case number being assigned to each patient and recorded in spreadsheets using Microsoft Excel® software (Microsoft Corporation, Redmond, WA). We carried out statistical analysis using the PSPP® software (GNU PSPP (Version 0.8.5) (Computer > Software). Boston, MA), where we performed hypothesis tests in comparison groups (student's t-test, analysis of variance (ANOVA) in addition to descriptive sample statistics. Regarding the study of potential correlations, we used Pearson's r correlation, Kendall rank correlation, and Spearman rank correlation. We assumed a value of statistical significance as p<0.05.

All required ethical procedures were followed, preserving the privacy of the data accessed in the clinical records of individuals involved in this study. This study was reviewed and accepted by the Ethical Commission for Health of Centro Hospitalar Universitário Cova da Beira under study number 51/2018 on 2018/10/10.

## Results

As shown in Table 2, our study population was characterized by a mean age of 82.7 years, consisting of 30% male and 70% female individuals. The mean hospitalization duration observed was 10.7 days while the cumulative readmission rate was 28% at the six-month follow-up. The male population presented a lower average age and mortality rate in the index hospitalization event, but their cumulative mortality after a six-month follow-up was higher (23%). Contrarily, female individuals, with an average age of 84.14 years, observed a tendency for a shorter hospital stay (10.4 days), and after six months of follow-up, a lower rate of readmission (24%) and death (20.88%). These observed differences were not found to be of any statistical significance.

**Table 2:** Demographic characterization of our study population Global and gender distribution of mean values of age, age of death, hospital stay duration, readmission, and mortality rates up to the six-month follow-up (n= 130)

	Number of cases	Age (years)	Hospitalization duration (days)	Readmission after 6-months	Inpatient Mortality	Cumulative 6-month Mortality	Mean Age of death (years)
Male	39	79.46	11.31	36%	10%	23.08%	88.42
Female	91	84.14	10.40	24%	14%	20.88%	85.11
Global	130	82.74	10.67	28%	13%	21.54%	87.36

The principal cause of hospital re-admission was a recurrence of respiratory infection, responsible for 58% of these events, of which, 30.5% were classified as new episodes of CAP, as occurring 90 days after the initial admission episode. Additionally, 16% were readmitted for decompensation of chronic heart failure, assuming its role as the second major cause of readmission after an initial CAP hospitalization event.

**Figure 1:**
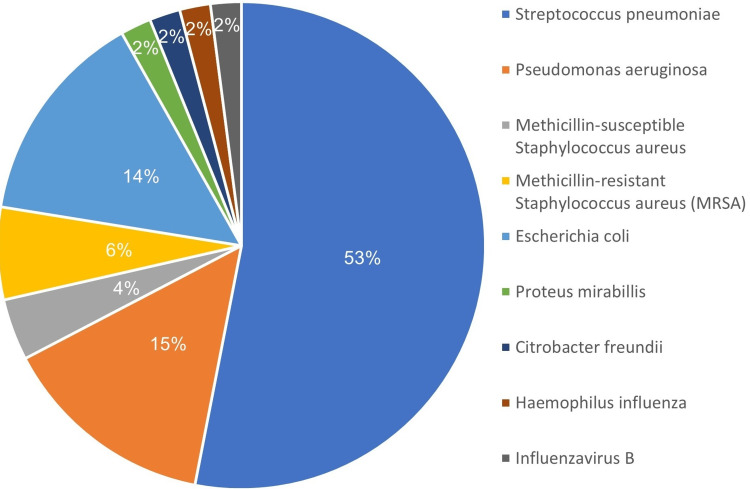
Microbiologic isolates on episodes of hospitalization by community-acquired pneumonia Representing case distribution per agent identified. Data from cases with successful identification of causative agent, corresponding to 48 patients or 36.92% of the total.

Identification of the etiological agent was achieved in only 48 cases, or 36.92% of the total sample. As seen in Figure 1, the most frequent agent was Streptococcus pneumoniae, corresponding to 54.17% of total positive cultures, with four cases being classified as invasive pneumococcal disease. Only one case of bacterial co-infection was observed, and no reported cases of viral and bacterial co-infection.

The CURB-65 score on admission was used to allocate the studied individuals into five subgroups according to their risk stratification (Table 3). Only one individual was attributed a CURB-65 score of 0, corresponding to 0.77% of patients. This single hospitalization event lasted five days, with no record of subsequent death or readmission during the follow-up period.

**Table 3:** Data distribution by CURB-65 scoring on hospital admission Presentation and comparison of variable data between patient subgroups obtained after application of CURB-65 score (5 subgroups – CURB = 0,1,2,3,4). Analysis of statistical significance (p-value) of difference observed between these subgroups.

	CURB-65 score on admission					
	0	1	2	3	4	P Value
Cases (%)	1 (0.77%)	3 (2.31%)	46 (35.38%)	68 (52.31%)	12 (9.23%)	--
Mean age (years)	33	67	81.07	84.26	88.58	p = 0.01
Mean hospitalization duration	5	6.67	11.76	10.06	11.42	p = 0.503
Inpatient mortality (%)	0 (0%)	0 (0%)	2 (4.35%)	11 (16.18%)	4 (33.33%)	p = 0.07
30-day mortality (%)	0 (0%)	0 (0%)	3 (6.52%)	11 (16.18%)	5 (41.67%)	p = 0.035
30-day mortality (reference)^18^ (%)	0 – 0.6%	0 – 3%	6.1 – 9.2%	13 – 21.4%	17 – 40%	--
90-day mortality (%)	0 (0%)	0 (0%)	4 (8.7%)	12 (17.65%)	7 (58.33%)	p = 0.01
6-month mortality (%)	0 (0%)	0 (0%)	6 (13.04%)	15 (22.06%)	7 (58.33%)	p = 0.012
Cumulative 6-month readmission rate (%)	0 (0%)	1 (33.33%)	11 (23.91%)	21 (30.88%)	3 (25%)	p = 0.892
Nosocomial infections (%)	0 (0%)	0 (0%)	10 (21.74%)	13 (19.12%)	4 (33.33%)	p = 0.838

Three cases (2.31% of the total) scored 1 point on the CURB-65 scale, averaging 67 years of age and 6.67 days of hospital stay. No deaths were reported in this subgroup, with only one occurrence of hospital readmission for a new occurrence of CAP.

The third group (CURB-65 = 2) accounted for 35.38% of the sample. This subgroup was characterized by a mean age of 81.07 years, and an average hospital stay of 11.76 days. The hospital mortality rate observed was 4.35%, gradually increasing to 6.52% and 8.7% at 30 and 90 days, respectively. The mortality rate after six months was reported as 13.04%, with a need for readmission occurring in 23.91% of these patients.

The majority of our patients (52.31%) were attributed 3 points on the CURB-65 scale. This moderate high-risk subgroup represented individuals with a mean age of 84.26 years and on average 10.06 days of hospitalization. The initial inpatient mortality rate of 16.18% gradually progressed to 22.06% at six months. Hospital readmission occurred in 30.88% of these cases.

The highest risk group (CURB-65 = 4) consisted of only 12 individuals (9.23% of the total) with a mean age of 88.58 years and a mean hospital stay of 11.42 days. Up to one-third of these patients would ultimately die during this initial hospitalization, a value that gradually increases on the subsequent follow-up, with a cumulative mortality rate and readmission rate of 58.33% and 25% after six months.

Between these subgroups, only the observed differences in age and mortality rate at 30 days, 90 days, and six months were found to be of statistical relevancy (p < 0.05). Comparing only the higher risk groups, (CURB ≥ 2), all these findings with the exception of the age variable preserved their described statistical significance. Additionally, all nosocomial infections occurring during the initial hospitalization episode were only observed in these higher-risk groups.

**Figure 2:**
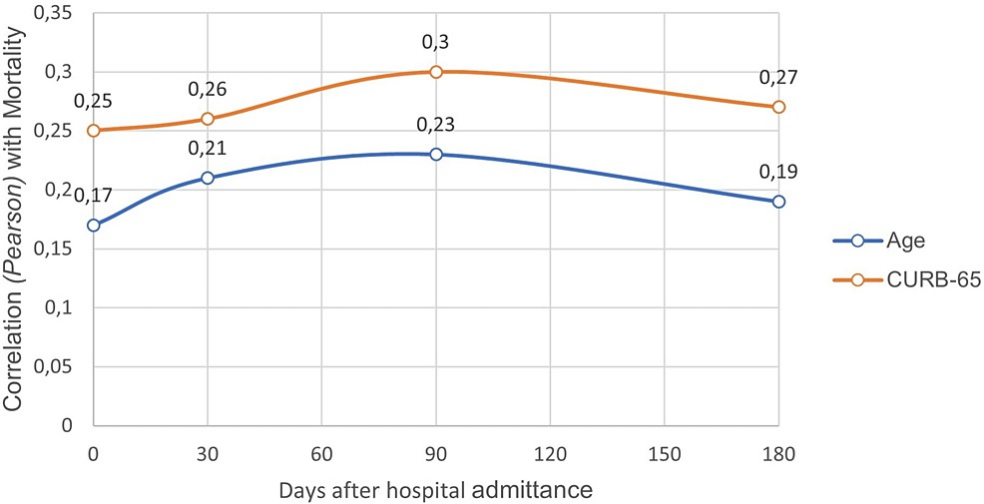
Correlation of observed mortality with CURB-65 score on admittance and individual age Comparison of the correlation coefficient (Pearson) between observed mortality rate (inpatient and 30,90 and 180 days after admittance) and either age or value obtained on the CURB-65 score on hospital admittance.

The CURB-65 score at admission along with individual age were both associated with a positive and statistically significant linear correlation with reported mortality rate, during the inpatient period as well as at 30 days, three months, and six months after admission. As observed in Figure 2, our data suggest that this correlation is more substantial with the CURB-65 score in opposition to the use of age as the only prognostic variable. There was also evidence of a positive correlation with statistical significance between the duration of the hospitalization event and the occurrence of nosocomial infections and readmissions.

## Discussion

The data obtained in this study refer to an aged population, with a great majority of individuals over 75 years old, corresponding to a mean age of 82.74 years. A higher value than expected in comparison with similar studies conducted previously in Portugal: 73.1 [12], 74.1 [22], and 79.1 years [21]. This relevant age factor could be assumed as the main bias factor of our study.

The rehospitalization rate after six months was 28%, well above the 14% described in the national 2014 data [10], but similar to the findings mentioned in other European data (8% to 46%) [9]. Most of these cases were found to correspond to a new episode of respiratory infection, demonstrating the frailty and relative immunosuppression status apparent in this age group.

Cultural identification of causative agents was achieved in only 36.92% of cases, a value near the lower limit of those reported on data from other European countries [2]. This could be explained by the fact that blood cultures were not conducted in all patients, according to clinical decisions, and that sputum specimens of satisfactory quality for laboratory processing were difficult to obtain in our population group. This insufficient efficacy of current processing techniques reinforces the need to develop alternative methods to achieve greater success in identifying microbiologic agents and potentiate more frequent targeted antibiotic use, improving prognosis and reducing health costs in infectious entities.

As expected, the predominant agent identified was Streptococcus pneumoniae, yet in a much higher proportion than reported in European (37-48%) [4,17] and in previous Portuguese studies (33-37%) [21-22]. This finding supports the historical and current prominent role of this microorganism in the pathological process of CAP and the importance of essential preventive measures such as anti-pneumococcal vaccination.

Contrary to initial expectations, there was no evidence of any correlation between the occurrence of nosocomial infections and mortality during initial admission or on later readmission. Thus, these might be considered a less relevant prognostic factor in either short- or long-term recovery, leading to the necessity for further research on the characterization of alternative, and more significant, factors implicated in the prognosis and risk stratification of these patients.

The inpatient mortality rate observed was 13%, lower than the national reported results in 2014 (26%) [10] and 2000-2009 (20.4%) [12], but slightly higher than that reported in E.U. countries (8.54%) [4]. Additionally, the 30-day mortality rate was also higher than similar to previous national studies (13.4% and 12.5%) [21-22], possibly accentuated by the older population found in our study. The verified mortality on the remaining cut-off periods: three and six months was found to be similar to that reported in previous similar studies [5,9,14].

The progressive increase in mortality rate maintained six months after an event of CAP hospitalization, from 14.62% at 30 days to 17.69% at three months and 21.54% at six months, highlights the less recognized long-term impact of a CAP hospitalization that was previously suggested in recent studies [5,6,14] that demonstrate an increased risk of all-cause death in comparison to the rest of the population in the same age group, even after two years [18].

The 30-day mortality rate demonstrated in the different risk groups was similar to what was mentioned in the initial study proposing the application of the CURB-65 scale, as well as with the findings in subsequent studies [19-20], validating its applicability and predictive value in the population covered by this hospital.

According to the proposed mortality risk stratification [18,20], only four individuals (3.08%) were classified as low mortality risk (CURB ≤ 1), with no formal indication for admission to inpatient care. This minimal percentage of low-risk patients hospitalized leads to the conclusion that, in the majority of our patients, the clinical decision about the treatment of CAP was supported by the application of the CURB-65 scale, with an adequate selection of high-risk patients.

Furthermore, 35.38% of individuals were attributed a moderate risk (CURB =2) while a large majority (61.54%) represented high mortality risk (up to 22%) with a CURB score equal to or higher than 3 [20]. There were no reported cases of CURB-65 scores higher than 4, leading to the possibility that these higher-risk patients were admitted early to an intensive care unit or died before being admitted to a ward.

**Figure 3:**
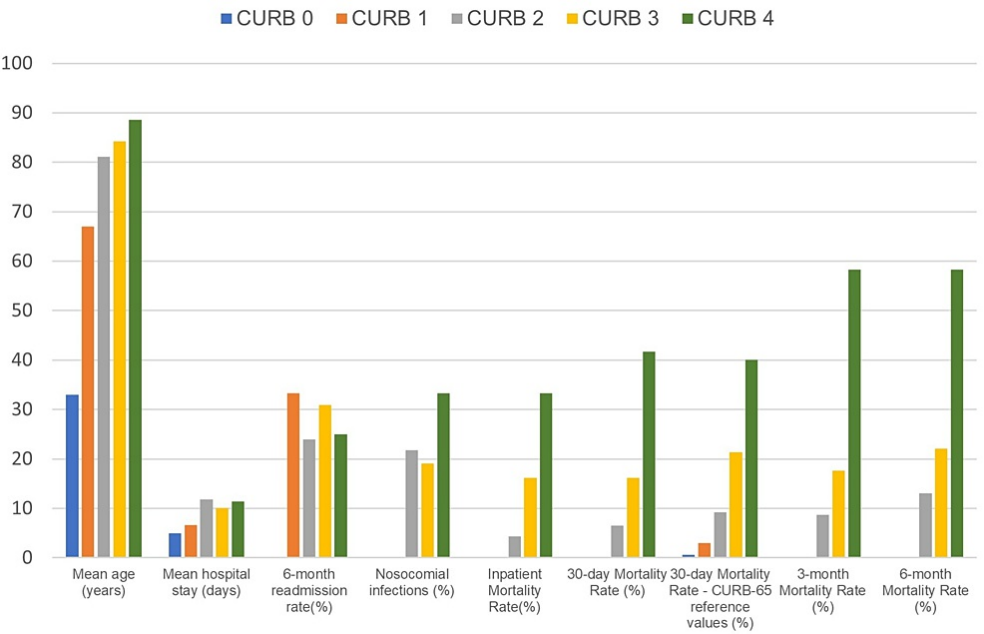
Variable data by subgroups based on the CURB-65 score on admission Mean age, hospital stay duration, readmission, nosocomial infection rates, and mortality data in relation to CURB-65 scoring. Observed variance of results between different subgroups.

As shown in Figure 3, higher values of CURB-65 at admission were associated with a progressive increase in mortality during the follow-up period. This moderate linear correlation between these two variables possesses statistical significance, preserved in all temporal cut-off points: during hospitalization and the six months after the primary CAP event. This reinforces the predictive potential of the CURB-65 scale, maintaining its usefulness in risk stratification and prognosis long after what was initially proposed and representing its potential assistance in identifying higher-risk patients that benefit from a more cautious and regular follow-up after a CAP episode.

As expected, a correlation between age at admission and mortality was also observed, however, this association was weaker and presented a less linear and lower predictive capacity. Also, the fact that there is no statistically significant difference in mean age among the higher-risk groups seems to indicate that the increased mortality reported in these groups, during these six months, is better predicted using the CURB-65 score than this age variable alone, demonstrating its importance as a tool of clinical utility.

This study demonstrates that the application of the CURB-65 score in a population admitted by CAP in a hospital center in Portugal is both adequate and reliable, even though there is a paucity of previous focused studies in our country about this question. Several notable limitations were identified in our study, notably: its relatively small sample size, a relevant potential bias associated with the significant high age variable in this population, and the fact that it only encompasses data from hospitalized patients, which limits the extrapolation of findings to the outpatient population.

## Conclusions

The CURB-65 score enables effective risk stratification with an important role in supporting clinical and therapeutic decision-making. Adequate identification of higher-risk patients remains one of the most important factors for personalized healthcare and subsequent improvement of prognosis and health results. Our results reinforce previous evidence of the existence of a gradual increase in long-term mortality after a CAP episode that should not be ignored and remains to be better characterized, to recognize other potential risk factors that could help identify those high-risk patients.

The data presented indicate that the CURB-65 score possesses not only the capacity to predict mortality and prognosis at 30 days after hospital admission, as initially proposed, but its usefulness might be extended to predict mortality at three and six months after a CAP episode. In our study, the higher risk subgroups, with a CURB-65 score of 3 and 4, presented a significant mortality rate after six month period, 22% and 58%, respectively. As expected, our findings support an association of longer episodes of hospitalization with an increase in the occurrence of nosocomial infections and the rate of readmission during a six-month follow-up.
